# Mitigating Spatio-Temporal Forgetting in Federated Continual Learning with Dynamic-Domain Clients

**DOI:** 10.3390/s26196273

**Published:** 2026-10-03

**Authors:** Dairao He, Ji Wang, Weidong Bao

**Affiliations:** National Key Laboratory of Big Data and Decision, National University of Defense Technology, Changsha 410073, China; hedairao_@nudt.edu.cn (D.H.); wdbao@nudt.edu.cn (W.B.)

**Keywords:** federated continual learning, catastrophic forgetting, prototype learning, dynamic client participation

## Abstract

Distributed sensing systems increasingly rely on geographically distributed devices that collect data under different operating conditions. Federated continual learning (FCL) enables a global model to be trained across distributed sensing clients whose local data evolve over time, while preserving previously acquired knowledge. Existing FCL studies have mainly addressed continual task evolution or class-based client heterogeneity, while federated domain-incremental learning has investigated adaptation to changing domains under a shared label space. Less attention has been paid to scenarios where continual task evolution and domain-shifted client participation occur simultaneously, leading to coupled temporal and spatial interference. In this paper, we study a dynamic-domain federated continual learning setting, where new clients share the same label space with source clients but introduce shifted feature distributions during training. This setting reveals a challenging form of spatio-temporal catastrophic forgetting: the model must learn evolving class-incremental tasks while preventing new-domain knowledge injection from degrading source-domain performance. To address this problem, we propose STP-FCL, a coordinated client–server framework. On the client side, STP-FCL preserves historical task semantics and source-domain discriminative structures through source-aware prototype-space optimization, reducing representation drift under evolving tasks and domains. On the server side, STP-FCL designs domain-projected aggregation to remove conflicting components from new-domain updates and adaptively control their fusion weight. Experiments under dynamic-domain federated continual learning scenarios show that STP-FCL achieves stronger source-domain knowledge retention and higher mixed-domain accuracy than representative baselines, with a trade-off in target-domain accuracy.

## 1. Introduction

**Background.** Modern networked sensing systems increasingly rely on distributed devices to collect and process data from different locations and operating environments. In recent years, federated learning (FL) has emerged as a distributed machine learning paradigm that enables multiple clients to collaboratively train a global model without moving their local data. During this process, participating clients perform local training and upload their model updates to a central server, which aggregates these updates to improve the global model [1]. By keeping data decentralized, FL provides an effective solution for privacy protection and reduces the cost of centralized data collection and communication [2,3]. It has shown broad application potential in sensor-related scenarios such as medical diagnosis [4,5] and intelligent transportation [6], as well as other distributed intelligent services such as financial risk control [7]. However, conventional FL usually assumes that the participating clients and their local data distributions remain relatively stable during training, making it difficult to handle open environments where data streams continuously evolve and clients participate dynamically. This work aims to support continual model updating in distributed sensing systems where the population of sensing devices and their acquisition conditions may evolve over time.

To improve the adaptability of FL in dynamic environments, recent studies have integrated FL with continual learning to formulate Federated Continual Learning (FCL). FedWeIT decomposes model parameters into global and task-adaptive components to support selective inter-client knowledge transfer [8], and GLFC tackles federated class-incremental learning through class-aware gradient compensation and class-semantic relation distillation [9]. These efforts relax the static-data assumption in conventional FL and enable models to learn from continuously evolving client data while retaining prior knowledge.

**Figure 1:**
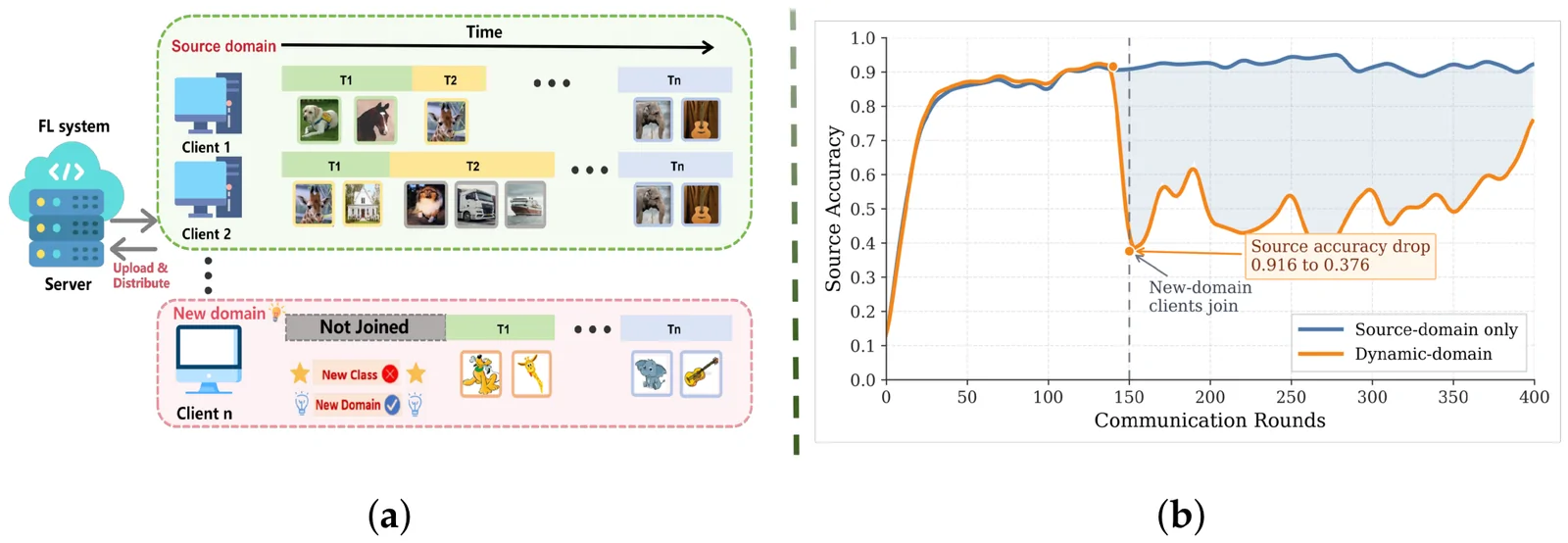
Motivation of dynamic-domain spatio-temporal federated continual learning. Using FedSpace as a representative baseline, source-domain-only training remains stable, whereas dynamic-domain participation causes a sharp source-domain accuracy drop after new-domain clients join. (**a**) Dynamic-domain FCL setting; (**b**) Source-domain degradation.

**Challenges.** Existing FCL studies have investigated catastrophic forgetting under temporal task evolution and heterogeneous federated learning from different perspectives. Recent spatio-temporal FCL methods further reveal that continual learning and federated heterogeneity may jointly affect knowledge retention. However, these formulations are mainly developed for class-incremental or private-class scenarios, where spatial heterogeneity is closely associated with differences in label spaces across clients. In parallel, federated domain-incremental learning considers changing input distributions under a shared label space and provides effective mechanisms for continual cross-domain adaptation. A more complex situation arises when these two forms of dynamics coexist. Clients continually progress through class-incremental task streams, while clients from shifted domains may also enter the federation and participate in global optimization. The former induces temporal representation drift as local tasks evolve, whereas the latter can introduce cross-domain update interference during server aggregation. Consequently, the global model must preserve previously acquired task knowledge while preventing domain-shifted updates from excessively perturbing the source-domain representations established before new-domain participation. For example, in multi-institution medical image analysis, differences in imaging devices and acquisition protocols across hospitals cause the same disease categories to exhibit diverse image styles. Figure 1 illustrates the studied dynamic-domain spatio-temporal FCL setting and provides empirical evidence for the resulting source-domain forgetting. Source-domain clients continuously learn evolving tasks, while new-domain clients join later with the same label space but shifted feature distributions. As shown on the right, introducing new-domain clients after the source-domain model becomes stable can sharply degrade source-domain accuracy, revealing the destructive interference caused by spatial domain increment.

**Solutions.** To explicitly address these dual challenges, we propose Spatio-Temporal Prototype-Preserved Federated Continual Learning (STP-FCL), a coordinated client–server framework designed to preserve semantic consistency across both dimensions. **To mitigate temporal forgetting caused by sequential task learning,** STP-FCL stabilizes client-side representations by employing feature-level prototype replay and contrastive learning to maintain historical semantic structures, while introducing Sharpness-Aware Minimization (SAM) to guide local models toward flatter, more generalizable minima. **To suppress spatial forgetting induced by domain-heterogeneous new clients,** the framework introduces a coordinated dual-guard mechanism. On the server side, domain-projected aggregation removes conflicting components from new-domain updates and adaptively controls their contribution to the global model. On the client side, source-domain prototype alignment constrains local class prototypes to remain close to the semantic anchors established before new-domain clients join.

**Contributions.** Our contributions can be summarized as follows:

- We formulate a dynamic-domain spatio-temporal FCL setting that combines continual class-incremental task evolution with the participation of domain-shifted clients under a shared label space. The formulation provides a unified framework for investigating source-domain knowledge retention when both the learned task sequence and the participating client domains evolve over time.
- We propose STP-FCL, a coordinated client–server framework that couples prototype-space semantic preservation during local continual learning with domain-projected aggregation at the server, jointly reducing temporal representation drift and cross-domain update interference.
- We evaluate STP-FCL on dynamic-domain visual recognition benchmarks that serve as controlled proxies for distributed sensing environments. The results demonstrate stronger source-domain retention and higher mixed-domain accuracy than representative baselines, while explicitly characterizing the trade-off between source-domain retention and target-domain adaptation.

## 2. Related Work

### 2.1. Federated Continual Learning

Federated continual learning (FCL) enables clients to learn from evolving local data streams while collaboratively maintaining a global model. Its key challenge is catastrophic forgetting under both temporal data evolution and federated heterogeneity [10,11]. Existing FCL methods mitigate forgetting through parameter isolation, distillation, replay, prototype sharing, or selective knowledge fusion. FedWeIT separates global and task-specific parameters for inter-client knowledge transfer [8]. In federated class-incremental learning (FCIL), GLFC uses class-aware gradient compensation and semantic relation distillation [9], while TARGET performs data-free server-side distillation with generated proxy samples [12]. FedProK transfers prototype-level feature knowledge [13], and FedSKF applies optimal transport for selective knowledge fusion [14]. These methods are effective in class-incremental settings, but they assume that new tasks or clients introduce new labels, and thus focus on old-class preservation rather than old-domain degradation under a shared label space.

Recent studies have explicitly considered spatio-temporal forgetting in FCL. FedCBC shows that temporal forgetting from sequential task learning and spatial forgetting from federated aggregation are coupled in FCIL, and mitigates them through class-specific binary classifiers, continual personalization, and selective fusion [15]. FedTA further analyzes spatio-temporal data heterogeneity as parameter- and output-forgetting, and stabilizes the feature space with Tail Anchors and global prototype selection [16].

### 2.2. Federated Domain-Incremental Learning

Domain-incremental learning considers continual learning with a fixed label space but changing input distributions. In federated settings, this problem is further affected by privacy constraints, client heterogeneity, communication limits, and dynamic participation. Recent federated domain-incremental learning (FDIL) methods address this problem from different perspectives. pFedDIL performs personalized adaptive knowledge matching by selecting relevant historical models for each new task [17]. RefFiL combines distillation, parameter regularization, and domain adaptation for replay-free FDIL [18]. SR-FDIL uses collaborative replay to coordinate cached samples across clients [19]. In privacy-sensitive applications such as digital pathology, continual domain-incremental learning has also been used to handle institutional and acquisition-related domain shifts [4].

Existing FDIL methods provide effective mechanisms for continual adaptation to evolving domains under a shared label space. For example, RefFiL considers federated domain-incremental learning with newly introduced domain information and develops a rehearsal-free adaptation mechanism, while SR-FDIL incorporates replay to preserve knowledge across domain changes. These methods primarily characterize continual learning along the domain-evolution dimension. The setting considered here additionally incorporates continual class-incremental task evolution into the federated learning process. As local task streams and client-domain composition evolve concurrently, representation drift accumulated across sequential tasks interacts with domain-induced update interference during global aggregation. STP-FCL addresses this interaction through prototype-space constraints that stabilize historical and source-domain semantics during local optimization, together with domain-projected aggregation that regulates conflicting updates from new-domain clients.

## 3. Problem Definition

We consider a federated continual learning system consisting of a central server and a client population $M=M^{\mathrm{src}}\cup M^{\mathrm{tgt}}$, where $M^{\mathrm{src}}\cap M^{\mathrm{tgt}}=\emptyset$. The server maintains a global model $\theta^{t}$ at communication round *t*. Source-domain clients $M^{\mathrm{src}}$ are available from the beginning of training, whereas target-domain clients $M^{\mathrm{tgt}}$, whose data follow a shifted input distribution, become available at a designated round $t_{0}$. Accordingly, the available client pool at round *t* is defined as

(1) $$A^{t}=\left\{\begin{array}{cc} M^{\mathrm{src}}, & t<t_{0},\\ M^{\mathrm{src}}\cup M^{\mathrm{tgt}}, & t\geq t_{0}. \end{array}\right.$$

At each communication round, the server samples a participating subset $S^{t}\subseteq A^{t}$ for local training and model aggregation. Thus, $A^{t}$ denotes the clients that have joined the federation by round *t*, whereas $S^{t}$ denotes the clients selected to participate in the current round. Each client owns a private local data stream and cannot share raw data with the server or other clients.

Each client follows a sequence of class-incremental tasks

(2) $$T_{m}=\left\{T_{m}^{1},T_{m}^{2},\ldots ,T_{m}^{K_{m}}\right\},$$

where $T_{m}^{k}$ denotes the *k*-th local task of client *m*. Let $Y_{m}^{k}\subseteq Y$ denote the class subset associated with $T_{m}^{k}$, and let $k_{m}\left(t\right)$ denote the latest task reached by client *m* at round *t*. The cumulative label set observed by client *m* up to round *t* is defined as

(3) $$Y_{m}^{\leq t}=\overset{k_{m}\left(t\right)}{\underset{k=1}{⋃}}Y_{m}^{k}.$$

Therefore, clients are sequentially exposed to new class subsets as their local task streams evolve, while previously encountered classes remain part of their learned knowledge. The shared-label assumption applies to the global label universe across domains rather than to the class subset observed in each individual task. Specifically, source and target domains share the same label semantics, while their conditional input distributions may differ:

(4) $$Y^{\mathrm{src}}=Y^{\mathrm{tgt}}=Y,\;P_{\mathrm{src}}\left(x|y\right)\neq P_{\mathrm{tgt}}\left(x|y\right),\;y\in Y.$$

Thus, class availability changes over time at the client level because of sequential task learning, whereas domain variation arises from differences in the input distributions and from changes in the participating client population. The two domains share the same label universe and do not introduce different label meanings.

At round *t*, the population-level federated risk over all clients that are currently available is defined as

(5) $$L_{A}^{t}\left(\theta \right)=\sum_{m\in A^{t}}\pi_{m}^{t}E_{\left(x,y\right)∼D_{m}^{\leq t}}\left[\ell (f(x;\theta ),y)\right],$$

where $D_{m}^{\leq t}$ denotes the data distributions encountered by client *m* up to round *t*. Following sample-size-weighted federated aggregation, the contribution weight of client *m* is defined as

(6) $$\pi_{m}^{t}=\frac{n_{m}^{t}}{\sum_{j\in A^{t}}n_{j}^{t}},$$

where $n_{m}^{t}$ denotes the number of local training samples associated with client *m* at round *t*. In an actual communication round, only the sampled clients $S^{t}\subseteq A^{t}$ perform local training. Accordingly, the round-level objective used for aggregation is approximated by the sampled-client empirical risk

(7) $${\hat{L}}_{S}^{t}\left(\theta \right)=\sum_{m\in S^{t}}{\hat{\pi }}_{m}^{t}{\hat{E}}_{\left(x,y\right)∼D_{m}^{\leq t}}\left[\ell (f(x;\theta ),y)\right],$$

where the normalized aggregation weight of a participating client is

(8) $${\hat{\pi }}_{m}^{t}=\frac{n_{m}^{t}}{\sum_{j\in S^{t}}n_{j}^{t}}.$$

When target-domain clients become available, their updates may affect the performance previously established on the source domain. We refer to this degradation as spatial forgetting. In the experiments, source-domain accuracy before and after target-domain participation is reported to characterize this effect, while the main comparison is based on source-domain, target-domain, and mixed accuracy.

Spatial forgetting refers to the instantaneous degradation of source-domain knowledge caused by the participation of target-domain clients. Let $A_{\mathrm{src}}\left(t_{0}^{-}\right)$ denote the source-domain accuracy of the global model at the last evaluation point before target-domain clients join, and let $A_{\mathrm{src}}\left(t_{0}^{+}\right)$ denote the source-domain accuracy at the first evaluation point after their participation begins. For the primary synchronized-arrival setting, we define the instantaneous spatial forgetting as

(9) $$F_{\mathrm{spa}}={\left[A_{\mathrm{src}}\left(t_{0}^{-}\right)-A_{\mathrm{src}}\left(t_{0}^{+}\right)\right]}_{+}.$$

A larger $F_{\mathrm{spa}}$ indicates a greater immediate degradation of source-domain performance after target-domain participation, and the value is reported in percentage points. For the staggered-participation setting, the same measure is computed between the source-domain accuracy immediately before the first target-domain client cohort joins and the accuracy at the first evaluation point after that cohort participates.

The learning goal is to adapt the global model to the evolving task and domain distributions while retaining previously acquired source-domain knowledge. STP-FCL addresses this goal through prototype-space semantic constraints during local continual learning and conflict-aware aggregation after target-domain participation. The experiments evaluate the resulting behavior using source-domain, target-domain, and mixed accuracy, while $F_{\mathrm{spa}}$ directly quantifies the instantaneous source-domain degradation caused by target-domain participation.

## 4. Motivation

### New-Domain Participation Induces Source Prototype Drift

We select communication round 150 as the source-domain anchoring point because, before this round, the source-domain model had entered a stable high-performance regime. At this stage, the source-domain accuracy has reached a stable level, and the learned class prototypes provide a reliable representation of the source-domain semantic structure. Therefore, the global source-domain prototypes recorded at the last communication round before target-domain participation, namely $t_{0}-1$, are used as the source-domain class anchors.

The source-domain accuracy drop after new-domain participation, shown in Figure 1, establishes the performance-level effect of dynamic-domain interference. To further examine its representation-level mechanism, we track whether the class prototypes of source-domain clients remain close to their anchors after round 150. Let $p_{m,c}^{t}$ denote the prototype of class *c* computed from source client *m* at communication round *t*, and let $a_{c}^{\mathrm{src}}$ denote the corresponding source-domain anchor recorded at round $t_{0}-1$. We quantify prototype drift using the cosine distance between each source-domain prototype and its corresponding anchor:

(10) $$D_{\mathrm{anchor}}^{t}=\frac{1}{|M^{\mathrm{src}}|\;C}\sum_{m\in M^{\mathrm{src}}}\sum_{c=1}^{C}\left(1-\frac{{\left(p_{m,c}^{t}\right)}^{⊤}a_{c}^{\mathrm{src}}}{{∥p_{m,c}^{t}∥}_{2}{∥a_{c}^{\mathrm{src}}∥}_{2}}\right).$$

where $M^{\mathrm{src}}$ denotes the set of source-domain clients, *C* is the number of classes, $p_{m,c}^{t}$ is the prototype of class *c* computed by source-domain client *m* at round *t*, and $a_{c}^{\mathrm{src}}$ is its corresponding source-domain anchor. Here, ${\left(\cdot \right)}^{⊤}$ denotes transpose and ${∥\cdot ∥}_{2}$ denotes the Euclidean norm. Each summand is the cosine distance between a class prototype and its anchor.

**Figure 2:**
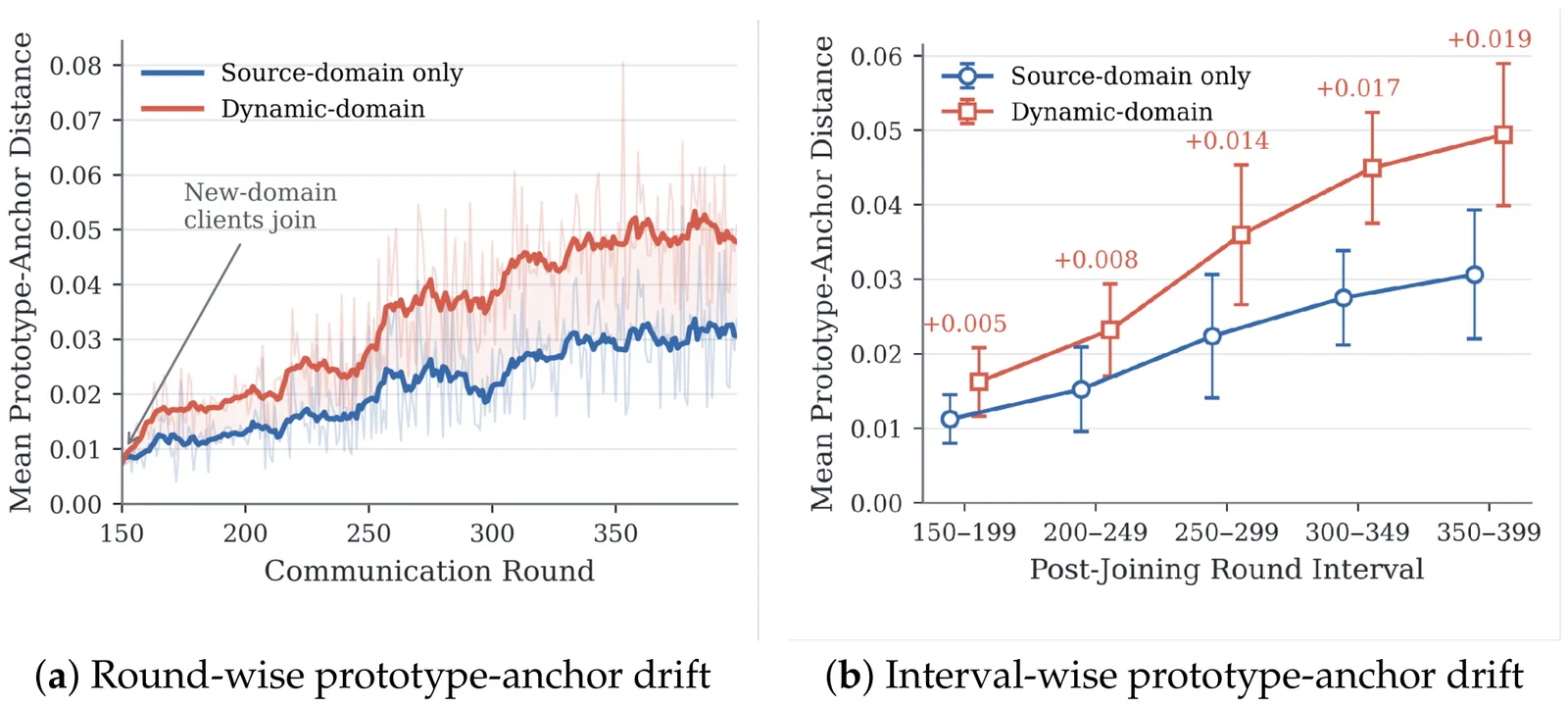
Illustrative single-run analysis of prototype-anchor drift using FedSpace with random seed 2002, comparing source-domain-only training with dynamic-domain training. Target-domain clients join at round $t_{0}=150$, and source-domain prototype anchors are recorded at round $t_{0}-1$. In panel (**a**), faint curves show the original round-wise measurements, while bold curves show a centered moving average over 10 communication rounds. In panel (**b**), each marker represents the mean prototype-anchor distance within a consecutive 50-round interval, and each error bar represents the sample standard deviation of measurements within that interval. These error bars describe within-run temporal variation, not uncertainty across independent runs. This single-run comparison is illustrative and does not establish statistical significance.

Figure 2 presents an illustrative comparison using FedSpace with random seed 2002. In this run, prototype-anchor distance increases gradually under source-domain-only training, whereas dynamic-domain training exhibits a larger distance after target-domain clients join. The gap between the interval means increases from 0.005 during rounds 150–199 to 0.019 during rounds 350–399.

The observed trajectories illustrate how source-domain representations may drift from their pre-joining anchors during dynamic-domain training. This example motivates preserving source-domain prototype structure when incorporating updates from newly participating domains.

## 5. Method

### 5.1. Overview

**Figure 3:**
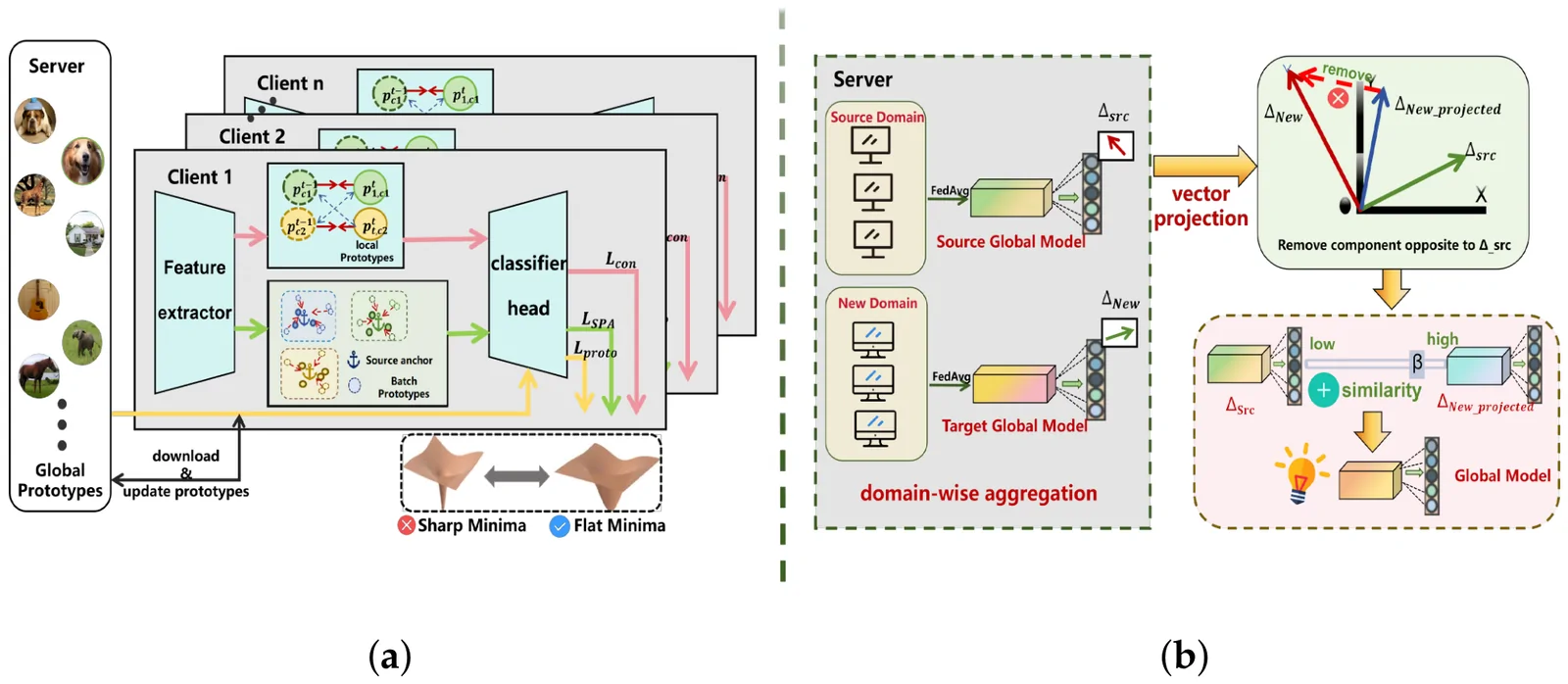
Overall framework of STP-FCL. Client-side prototype-space optimization preserves historical and source-domain semantics, while server-side domain-projected aggregation suppresses conflicting new-domain updates before global model fusion. (**a**) Client-side prototype optimization; (**b**) Server-side projected aggregation.

Inspired by the above empirical discoveries, we propose Spatio-Temporal Prototype-Preserved Federated Continual Learning(STP-FCL). The central idea of STP-FCL is to preserve semantic consistency across both local optimization and global aggregation. Here, semantic consistency means that samples with the same class semantics should remain close in the prototype space across different tasks, domains, and communication rounds, while global aggregation should avoid destroying such source-domain semantic structures. Based on this principle, prototype-space constraints reduce client-side representation drift, and domain-projected aggregation suppresses destructive interference from new-domain updates. Through this coordinated client–server design, STP-FCL enables the global model to learn evolving tasks and adapt to new domains without severely degrading previously learned source-domain knowledge. The overall framework is illustrated in Figure 3 (see Algorithm 1 for the complete algorithm).

On the client side, STP-FCL performs source-preserved prototype-space optimization, which uses prototype-guided local training to preserve historical task semantics and stabilize cross-domain representations. This reduces temporal drift from sequential tasks and spatial drift from new-domain data. Sharpness-aware minimization (SAM) is adopted to obtain flatter local solutions and improve aggregation stability under heterogeneous continual learning. On the server side, we maintain global prototypes, capture source-domain prototype anchors before new-domain participation, and introduce domain-projected aggregation to remove the conflicting component of new-domain updates before fusing them with source-domain updates.
**Algorithm 1** Training Procedure of STP-FCL**Require:** Initial global model $\theta^{0}$, source clients $M^{src}$, new-domain clients $M^{tgt}$, joining round $t_{0}$, total rounds *T*
  1:Initialize global prototype memory $P^{0}$  2:**for** t=0,1,…,T−1 **do**  3:      Sample participating clients $S^{t}\subseteq A^{t}$  4:      Server sends $\theta^{t}$ and global prototypes $P^{t}$ to selected clients  5:      **if** $t=t_{0}-1$ **then**  6:            Store source-domain anchors $A^{src}={\left\{a_{c}^{src}=p_{c}^{t_{0}-1}\right\}}_{c\in Y}$  7:      **end if**  8:      **for** each selected client $m\in S^{t}$ **do in parallel**  9:         Receive $\theta^{t}$, $P^{t}$, and $A^{src}$ if available10:         Construct prototype replay samples from global prototypes11:         Compute $L_{\mathrm{CE}}$, $L_{\mathrm{proto}}$, $L_{\mathrm{con}}$, and $L_{\mathrm{SPA}}$12:         Update local model with SAM on$$L_{m}^{t}=L_{\mathrm{CE}}+\lambda_{p}L_{\mathrm{proto}}+\lambda_{c}L_{\mathrm{con}}+L_{\mathrm{SPA}}$$13:         Upload local model $\theta_{m}^{t+1}$ and local class prototypes to the server14:      **end for**15:      Update global prototype memory $P^{t+1}$ using uploaded local prototypes16:      **if** $t<t_{0}$ **then**17:         Aggregate selected source-domain clients by FedAvg to obtain $\theta^{t+1}$18:      **else**19:         Separately aggregate $S_{src}^{t}$ and $S_{tgt}^{t}$ to obtain $\theta_{src}^{t+1}$ and $\theta_{tgt}^{t+1}$20:         Compute updates $\Delta_{src}^{t+1}=\theta_{src}^{t+1}-\theta^{t}$ and $\Delta_{tgt}^{t+1}=\theta_{tgt}^{t+1}-\theta^{t}$21:         **if** $\langle \Delta_{tgt}^{t+1},\Delta_{src}^{t+1}\rangle <0$ **then**22:           Remove the conflicting component of $\Delta_{tgt}^{t+1}$ along $\Delta_{src}^{t+1}$23:         **end if**24:         Compute similarity-adaptive fusion weight $\beta^{t+1}$25:         Fuse $\theta_{src}^{t+1}$ and projected ${\tilde{\theta }}_{tgt}^{t+1}$ to obtain $\theta^{t+1}$26:      **end if**27:**end for**28:**return** Final global model $\theta^{T}$


From a distributed sensing perspective, source-domain clients represent established sensing devices or locations, whereas target-domain clients represent newly deployed devices operating under different acquisition conditions. The source-preserved prototype alignment maintains the semantic structure learned from established sensing devices, while domain-projected aggregation controls the influence of updates from newly introduced sensing domains. This client–server coordination enables the global model to incorporate new sensing observations without excessively degrading previously acquired source-domain knowledge.

### 5.2. Client-Side Prototype-Space Optimization

For client *m* at communication round *t*, the local objective is defined as:

(11) $$L_{m}^{t}=L_{\mathrm{CE}}+\lambda_{p}L_{\mathrm{proto}}+\lambda_{c}L_{\mathrm{con}}+L_{\mathrm{SPA}},$$

Following SAM [20,21], we optimize this objective with a flatness-aware local update, adapting it to the federated continual learning scenario to improve the robustness and aggregability of client updates under task and domain heterogeneity, where $L_{\mathrm{CE}}$ is the standard classification loss, $L_{\mathrm{proto}}$ is the prototype replay loss, $L_{\mathrm{con}}$ is the contrastive representation loss, and $L_{\mathrm{SPA}}$ is the source-preserved prototype alignment loss. The first two regularization terms address temporal forgetting in continual task learning [21,22], while $L_{\mathrm{SPA}}$ targets spatial forgetting induced by new-domain client participation.

Although this objective contains multiple regularization terms, they are not independent or conflicting constraints. All three anti-forgetting terms operate in the same prototype space but preserve different aspects of the model. The prototype replay loss preserves historical classifier boundaries, the contrastive loss maintains class-discriminative feature geometry, and the SPA loss prevents source-domain prototype drift under new-domain participation. Thus, temporal and spatial forgetting are addressed through a unified semantic representation rather than through unrelated penalties.

#### 5.2.1. Temporal Prototype Replay and Contrastive Learning

To alleviate temporal forgetting, the server maintains global class prototypes from previous rounds [22]. Since raw historical data cannot be stored or exchanged in federated learning, each client replays historical knowledge by synthesizing virtual feature samples around global prototypes:

(12) $${\hat{p}}_{c}^{t}=p_{c}^{t-1}+r^{t-1}\epsilon ,\;\epsilon ∼N\left(0,1\right),$$

where $p_{c}^{t-1}$ is the global prototype of class *c* from the previous round and $r^{t-1}$ denotes the global feature dispersion radius. The prototype replay loss is computed by feeding the synthesized prototypes into the classifier:

(13) $$L_{\mathrm{proto}}=\frac{1}{|{\hat{P}}^{t}|}\sum_{\left(\hat{p},y\right)\in {\hat{P}}^{t}}\ell_{\mathrm{CE}}\left(D(\hat{p};\psi_{m}^{t}),y\right).$$

This term preserves historical classifier boundaries in the feature space without accessing raw old-task samples.

STP-FCL exchanges class-level feature prototypes rather than raw samples. This design reduces the exposure of instance-level data compared with direct data sharing, but it does not provide a formal privacy guarantee. Recent empirical work has examined feature-space hijacking attacks that attempt to reconstruct input data from uploaded prototypes and reported that salient image content could not be recovered even after extensive optimization [23]. Since the prototypes used in STP-FCL are class-level aggregated representations rather than individual sample features, reconstructing a specific raw sample may be more challenging. Nevertheless, protection against stronger inversion or membership-inference attacks would require dedicated privacy mechanisms, such as secure aggregation or differential privacy, which are beyond the scope of this work.

Previous works showed that contrastive learning can be efficiently used in continual learning to tackle catastrophic forgetting [24,25]. We further impose a contrastive representation loss on current features and sampled prototype features. Let $Z_{m}^{t}$ denote the set of features used for contrastive learning. For each feature $z_{i}$, its positives are features with the same class label and its negatives are features from different classes:

(14) $$L_{\mathrm{con}}=-\frac{1}{|Z_{m}^{t}|}\sum_{z_{i}\in Z_{m}^{t}}\frac{1}{\left|P(i)\right|}\sum_{z_{j}\in P\left(i\right)}\log\frac{\exp(\mathrm{sim}\left(z_{i},z_{j}\right)/\tau )}{\sum_{z_{k}\in Z_{m}^{t},k\neq i}\exp\left(\mathrm{sim}\left(z_{i},z_{k}\right)/\tau \right)}.$$

This loss encourages intra-class compactness and inter-class separation, stabilizing the semantic structure learned across sequential tasks.

#### 5.2.2. Spatial Source-Preserved Prototype Alignment

Temporal anti-forgetting alone is insufficient when new-domain clients join the federation, since new-domain updates may shift the shared representation space and damage source-domain decision structures. To mitigate this spatial forgetting, the server stores source-domain prototype anchors before new-domain clients start training:

(15) $$A^{src}={\left\{a_{c}^{src}\right\}}_{c\in Y},\;a_{c}^{src}=p_{c}^{t_{0}-1},$$

where $t_{0}$ denotes the communication round when new-domain clients join. During local training, client *m* first computes a batch-level prototype for each observed class *c* and then aligns its direction with that of the corresponding source-domain anchor. Let $B_{m,c}^{t}=\left\{\left(x,y\right)\in B_{m}^{t}:y=c\right\}$ denote the subset of samples belonging to class *c* in the local mini-batch. The batch-level prototype is computed as

(16) $${\bar{z}}_{m,c}^{t}=\frac{1}{|B_{m,c}^{t}|}\sum_{\left(x,y\right)\in B_{m,c}^{t}}f\left(x;\theta_{m}^{t}\right).$$

The source-preserved alignment loss is then defined using cosine distance:

(17) $$L_{\mathrm{spa}}=\frac{1}{|C_{m}^{t}|}\sum_{c\in C_{m}^{t}}\left(1-\frac{{\left({\bar{z}}_{m,c}^{t}\right)}^{⊤}a_{c}^{\mathrm{src}}}{{∥{\bar{z}}_{m,c}^{t}∥}_{2}{∥a_{c}^{\mathrm{src}}∥}_{2}}\right),$$

where $C_{m}^{t}$ denotes the set of classes observed by client *m* at round *t*. By minimizing the discrepancy between the current class prototypes and the source-domain anchors, $L_{\mathrm{spa}}$ preserves the source-domain semantic structure.

For source-domain clients, this loss anchors their representations to the pre-injection source prototype space, preventing source knowledge from drifting after new-domain participation. For new-domain clients, it is activated after a warm-up period and serves as a semantic alignment constraint to avoid excessive distortion of the shared class structure. Formally,

(18) $$L_{\mathrm{SPA}}=\left\{\begin{array}{cc} \lambda_{src}L_{\mathrm{spa}},\; & m\in M^{src},\;t\geq t_{0},\\ \lambda_{tgt}L_{\mathrm{spa}},\; & m\in M^{tgt},\;t\geq t_{0}+T_{w},\\ 0,\; & \mathrm{otherwise}, \end{array}\right.$$

where $\lambda_{src}$ and $\lambda_{tgt}$ control source anchoring and target-to-source alignment, respectively, and $T_{w}$ denotes the warm-up period for new-domain clients.

### 5.3. Server-Side Domain-Projected Aggregation

Although client-side prototype constraints reduce representation drift, server aggregation may still suffer from destructive interference when new-domain updates conflict with source-domain updates. We therefore design a domain-projected aggregation (DPA) strategy inspired by gradient conflict projection [26]. This strategy protects the source-domain update direction while still allowing compatible new-domain knowledge to be injected into the global model. Let

$$S_{src}^{t}=S^{t}\cap M^{src},\;S_{tgt}^{t}=S^{t}\cap M^{tgt}.$$

Specifically, the server first aggregates source-domain and new-domain clients separately:

(19) $$\begin{array}{cc} \theta_{src}^{t+1} & =\mathrm{FedAvg}\left(\{\theta_{m}^{t+1}:m\in S_{src}^{t}\}\right),\\ \theta_{tgt}^{t+1} & =\mathrm{FedAvg}\left(\{\theta_{m}^{t+1}:m\in S_{tgt}^{t}\}\right). \end{array}$$

Their updates from $\theta^{t}$ are:

(20) $$\Delta_{src}^{t+1}=\theta_{src}^{t+1}-\theta^{t},\;\Delta_{tgt}^{t+1}=\theta_{tgt}^{t+1}-\theta^{t}.$$

To suppress destructive interference from new-domain updates, we project the target-domain update when it conflicts with the source-domain update. Specifically, if the inner product between $\Delta_{tgt}^{t+1}$ and $\Delta_{src}^{t+1}$ is negative, the component of $\Delta_{tgt}^{t+1}$ that is opposite to the source update direction is removed; otherwise, the target update is kept unchanged. The corrected target update and the corresponding projected target model are defined as:

(21) $$\begin{array}{cc} {\tilde{\Delta }}_{tgt}^{t+1} & =\left\{\begin{array}{cc} \Delta_{tgt}^{t+1}-\frac{\langle \Delta_{tgt}^{t+1},\Delta_{src}^{t+1}\rangle }{∥\Delta_{src}^{t+1}{∥}_{2}^{2}}\Delta_{src}^{t+1},\; & \langle \Delta_{tgt}^{t+1},\Delta_{src}^{t+1}\rangle <0,\\ \Delta_{tgt}^{t+1},\; & \langle \Delta_{tgt}^{t+1},\Delta_{src}^{t+1}\rangle \geq 0, \end{array}\right.\\ {\tilde{\theta }}_{tgt}^{t+1} & =\theta^{t}+{\tilde{\Delta }}_{tgt}^{t+1}. \end{array}$$

After conflict projection, the server adaptively determines how much new-domain knowledge should be injected into the global model. We compute the cosine similarity between the original source-domain and target-domain update directions, and map it to a target-domain fusion weight through a sigmoid gate:

(22) $$\begin{array}{cc} s^{t+1} & =\frac{\langle \Delta_{src}^{t+1},\Delta_{tgt}^{t+1}\rangle }{{∥\Delta_{src}^{t+1}∥}_{2}{∥\Delta_{tgt}^{t+1}∥}_{2}},\\ \beta^{t+1} & =\beta_{\min}+\beta_{\mathrm{range}}\cdot \sigma \left(\frac{s^{t+1}}{\tau_{s}}\right), \end{array}$$

where σ(·) is the sigmoid function and $\tau_{s}$ is the similarity temperature; higher similarity allows a larger target-domain fusion weight.

The server then fuses the source-domain model and the projected target-domain model with the adaptive weight. When temporal model smoothing is enabled, the fused model is further averaged with the previous global model:

(23) $$\begin{array}{cc} \theta_{sp}^{t+1} & =\left(1-\beta^{t+1}\right)\theta_{src}^{t+1}+\beta^{t+1}{\tilde{\theta }}_{tgt}^{t+1},\\ \theta^{t+1} & =\omega \theta_{sp}^{t+1}+\left(1-\omega \right)\theta^{t}, \end{array}$$

where ω controls the update strength. This server-side procedure removes conflicting new-domain components, adaptively controls their influence, and allows the global model to absorb useful new-domain knowledge.

## 6. Experiments

### 6.1. Experimental Setup

**Datasets and Dynamic-Domain Scenario.** We evaluate STP-FCL on digit-based dynamic-domain FCL scenarios. MNIST [27] serves as the source domain, while SVHN [28] and SynthDigits (SYNTH) [29] are used as target domains with the same digit label space 0–9 but shifted visual distributions; SYNTH is subsampled to a scale comparable to SVHN.

The federation includes 20 source-domain clients and 10 target-domain clients. Source-domain clients participate from the beginning, whereas target-domain clients become available at communication round 150. The digit classes are divided into five class-incremental tasks with two classes per task. To introduce task-level heterogeneity, clients may follow different task orders and client-specific task-switching schedules, as described in Appendix A. The task stream of each target-domain client starts when that client joins the federation.

Client data are partitioned using a power-law Dirichlet strategy with concentration parameter α=4.0. The same partitioning procedure, client split, task-stream generation, and target-client joining protocol are used for all compared methods.

**Baselines.** We compare STP-FCL with seven representative baselines, including general federated continual learning methods, prototype-based asynchronous FCL, exemplar-free class-continual learning, and dedicated federated domain-incremental learning methods.

FedLwF [30] uses the previous global model as a teacher and applies local knowledge distillation to preserve previously learned predictions. Although it is frequently evaluated in class-incremental settings, its output-level distillation constraint is applicable to general FCL scenarios and does not require the arrival of new classes.

FedEWC [31] estimates the importance of model parameters for historical tasks and penalizes changes to important parameters during local training. As a parameter-regularization method, FedEWC is likewise not restricted to class-incremental learning and can be directly applied when client data distributions evolve over time.

Re-Fed [32] retains representative historical samples locally and replays them while learning new data. Its replay mechanism is label-space agnostic; therefore, it serves as a general replay-based FCL baseline under the dynamic-domain protocol.

FedSpace [22] is a prototype-based asynchronous FCL method that uses prototype replay and representation regularization to preserve historical knowledge under heterogeneous client task streams.

TagFed [33] performs task-aware federated aggregation by sharing the feature extractor across participating clients while grouping classifier updates according to the clients’ current tasks. It is adapted to the asynchronous shared-label dynamic-domain setting using the same client participation and task-stream protocol as the other baselines.

RefFiL [18] is a rehearsal-free federated domain-incremental learning method that combines a domain-adaptive prompt generator, global prompt learning, and domain-specific prompt contrastive learning to preserve domain-invariant knowledge.

SR-FDIL [19] is a replay-based federated domain-incremental learning method. It selects locally cached samples by combining domain-representative and cross-client collaborative importance scores, and further adopts importance-aware client selection.

All baselines are evaluated under the same dynamic-domain FCL protocol as STP-FCL, including the same client split, class-incremental task streams, target-domain joining round, backbone, local training budget, communication rounds, and evaluation procedure. For methods originally proposed for class-incremental learning, new-domain clients are allowed to join at the designated round while the shared label space is kept unchanged.

**Implementation Details.** We use ResNet-18 as the backbone model for all methods. The total number of communication rounds is 400, and 10 clients are sampled in each round. Each selected client performs 10 local epochs with a batch size of 64. The learning rate is set to 0.003. Target-domain clients start participating from round 150. Evaluation is conducted every 10 communication rounds.

For fair comparison, all compared methods follow the same backbone, task split, client partition, communication schedule, local training budget, optimizer setting, and evaluation protocol. For class-incremental baselines originally designed outside the federated continual learning setting, we preserve their original class-incremental learning mechanisms and integrate them into the same federated training pipeline. The baseline-specific hyperparameters used in our implementation are reported in Appendix E, Table A9, and are kept fixed across random seeds.

For STP-FCL, the prototype replay and contrastive loss weights are both set to $1\times 10^{-2}$. In SPA, we set the source anchoring weight to $\lambda_{src}=3.0$ and the target alignment weight to $\lambda_{tgt}=0.5$, with target-domain alignment activated after a 20-round warm-up following target-client participation. For domain-projected aggregation, we set $\beta_{\min}=0.35$, $\beta_{\mathrm{range}}=0.15$, and the similarity temperature to $\tau_{s}=0.5$. The base weight $\beta_{\min}=0.35$ ensures that newly joined clients retain a minimum contribution consistent with their approximately one-third participation proportion in the federation. The adaptive range $\beta_{\mathrm{range}}=0.15$ permits a larger contribution only when the target-domain update is highly compatible with the source-domain update. Consequently, the target-domain fusion weight is bounded above 0.50, preventing newly joined clients from dominating the global aggregation while preserving sufficient capacity for domain adaptation.

**Evaluation Metrics.** We report three metrics after new-domain clients join: source-domain average accuracy (Source AA), target-domain average accuracy (Target AA), and mixed-domain average accuracy (Mix AA). Since the model is evaluated every 10 communication rounds, we average the corresponding accuracy values over all evaluation rounds after the joining round $t_{0}$:

(24) $${\mathrm{AA}}_{d}=\frac{1}{|R_{post}|}\sum_{t\in R_{post}}A_{d}^{t},\;d\in \left\{src,tgt,mix\right\},$$

where $R_{post}$ denotes evaluation rounds after new-domain participation. Source AA evaluates retention on the source domain, Target AA measures adaptation to the new domain, and Mix AA reports the average accuracy over all observed classes across both domains. At each evaluation round, mixed-domain accuracy is computed as the total number of correct predictions across the source and target evaluation subsets divided by their combined number of samples. In our setting, Source AA and Mix AA are the primary metrics for assessing aggregate source-domain retention and overall predictive performance across the source and target domains under task evolution and new-domain participation.

### 6.2. Main Results and Analysis

**Figure 4:**
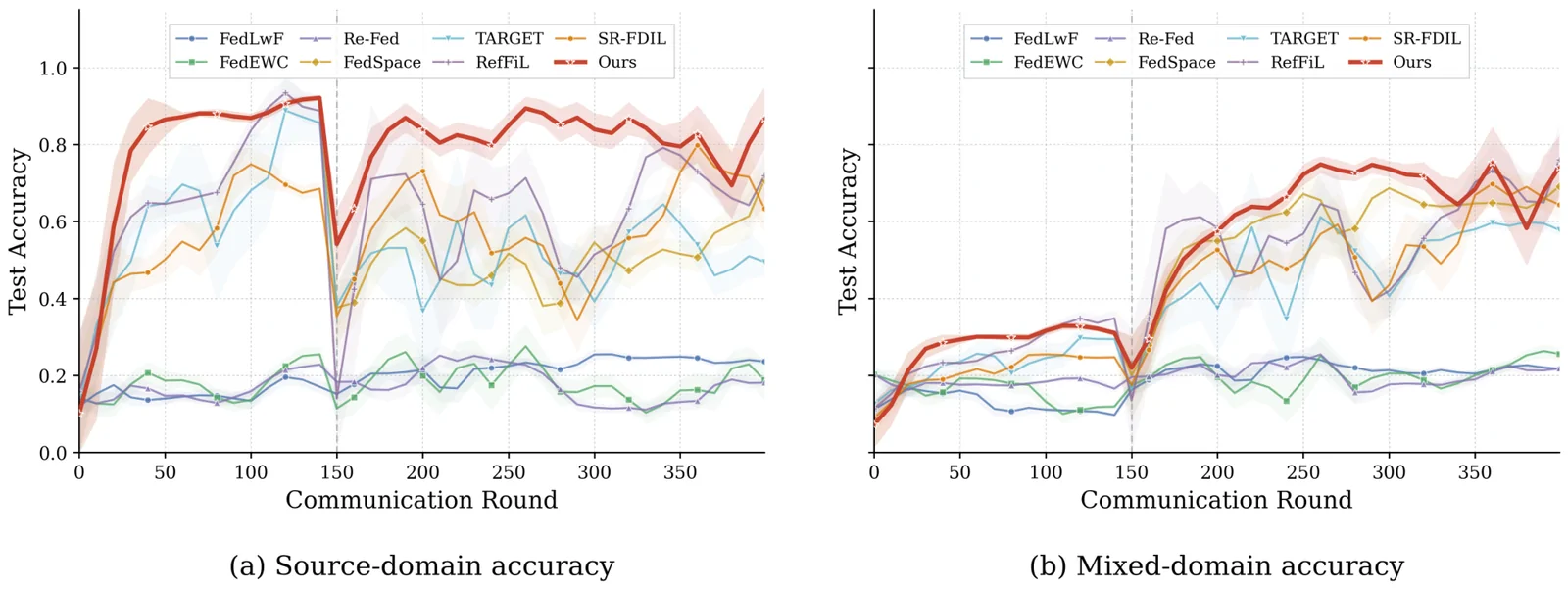
Accuracy curves on MNIST+SVHN under dynamic-domain federated continual learning.

Table 1 reports the main quantitative results on two dynamic-domain settings, MNIST+SVHN and MNIST+SynthDigits. All metrics are computed after new-domain clients join the federation. The best result is shown in bold and the second-best result is underlined. Figure 4 further visualizes the source-domain and mixed-domain accuracy curves over communication rounds.

**Source AA** STP-FCL achieves the best Source AA on both dynamic-domain settings. On MNIST+SVHN, STP-FCL improves Source AA from 62.95% to 81.36% compared with the strongest baseline. On MNIST+SynthDigits, STP-FCL improves Source AA from 84.63% to 87.31%. These results show that STP-FCL effectively mitigates source-domain degradation caused by new-domain client participation.

**Mix AA** STP-FCL also obtains the best Mix AA on both settings, reaching 64.05% on MNIST+SVHN and 78.96% on MNIST+SynthDigits. This indicates that the proposed method improves overall spatio-temporal learning performance rather than merely optimizing source-domain accuracy. As shown in Figure 4, STP-FCL maintains a more stable source-domain trajectory after the joining round and achieves stronger mixed-domain accuracy, suggesting that source-domain preservation contributes to a more reliable global model under dynamic-domain continual learning.

**Target AA** FedSpace achieves the highest Target AA on MNIST+SVHN, whereas SR-FDIL performs best on MNIST+SynthDigits. On MNIST+SVHN, STP-FCL obtains a Target AA of 57.40%, compared with 63.11% for FedSpace, while achieving a higher Source AA (81.36% versus 50.89%) and Mix AA (64.05% versus 59.72%). Similarly, on MNIST+SynthDigits, STP-FCL obtains a lower Target AA than SR-FDIL (70.21% versus 76.31%), but achieves higher Source AA (87.31% versus 70.93%) and Mix AA (78.96% versus 73.56%). These results reveal a retention–adaptation trade-off: stronger source-domain preservation comes at the cost of some target-domain accuracy, while yielding higher mixed-domain accuracy in both evaluated settings.

### 6.3. Ablation Study

Table 2 reports the mean ± sample standard deviation of Source AA, Target AA, and Mixed AA over three random seeds. The complete STP-FCL configuration achieves the highest mean Source AA (82.83±1.29%) and Mixed AA (64.87±0.96%) among the evaluated configurations. Removing prototype replay and contrastive learning, SPA, or SAM reduces mean Source AA to 80.33%, 80.25%, and 81.10%, respectively, and reduces mean Mixed AA to 63.48%, 63.45%, and 64.03%. These results are consistent with the contributions of historical prototype preservation, source-domain alignment, and flatness-aware local optimization under the evaluated protocol.

Removing DPA produces the largest reduction in mean Source AA, from 82.83% to 68.69%, while increasing mean Target AA from 57.98% to 61.62%. Mean Mixed AA nevertheless decreases from 64.87% to 63.58%. This pattern illustrates a retention–adaptation trade-off: DPA favors source-domain preservation at the cost of some target-domain accuracy, while improving mean mixed-domain performance in this experiment. The complete model therefore provides the highest mean source-domain and mixed-domain accuracies among these ablation configurations.

### 6.4. Parameter Sensitivity Analysis

We further investigate the sensitivity of STP-FCL to two key parameters on the MNIST+SVHN dynamic-domain setting: the source-domain anchoring weight $\lambda_{\mathrm{src}}$ in SPA and the fusion-strength scaling factor ρ in DPA. The latter scales the adaptive new-domain fusion weight in DPA, i.e., ${\tilde{\beta }}^{t}=\rho \beta^{t}$. Here, ρ is introduced only for sensitivity analysis, with the default setting ρ=1, and is not an additional learnable parameter of STP-FCL. We report Source AA and MixAA after new-domain clients join the federation.

**Effect of source-domain anchoring.** As shown in Table 3, STP-FCL achieves the best Source AA and MixAA when $\lambda_{\mathrm{src}}=3$. A smaller anchoring weight ($\lambda_{\mathrm{src}}=1$) provides insufficient constraints on source-domain prototype drift after new-domain participation. In contrast, an excessively large weight ($\lambda_{\mathrm{src}}=5$) restricts representation adaptation and also reduces both metrics. These results indicate that a moderate source-domain anchoring strength provides the most effective balance between source-domain preservation and continual adaptation.

**Effect of DPA fusion strength.** The DPA fusion scale controls the contribution of the projected new-domain update during global aggregation. When the fusion strength is reduced to 0.5×, STP-FCL obtains a higher Source AA of 85.62%, but its MixAA decreases substantially to 48.04%. This result indicates excessive protection of source-domain knowledge at the expense of new-domain adaptation. Conversely, increasing the fusion strength to 1.5× causes Source AA to decrease sharply to 64.13%, because overly strong new-domain updates introduce greater interference with the source-domain model. The default 1.0× setting achieves the best MixAA and maintains strong source-domain retention, supporting the selected DPA fusion configuration.

### 6.5. Computational and Communication Overhead

We further analyze the communication and computational overhead of STP-FCL and the compared methods, as shown in Table 4. Since all methods transmit the global model between the server and selected clients, the standard model exchange is shared by all methods and costs approximately 875.20 MiB per communication round when 10 clients are selected. Therefore, we focus on the method-specific extra payload beyond the standard model upload and download. Detailed local-training and server-side aggregation measurements are reported in Appendix B.

STP-FCL introduces only 1.02 MiB additional communication per round, which accounts for approximately 0.12% of the standard model-exchange cost. This overhead is lower than that of RefFiL and SR-FDIL, and remains negligible compared with the common communication cost required by federated training. In terms of computation, STP-FCL introduces additional local optimization through SAM and the SPA loss. Importantly, these operations do not require transmitting generators, discriminators, or large replay data. Thus, STP-FCL improves spatio-temporal robustness while maintaining a small communication overhead.

### 6.6. Implications for Distributed Sensing

The proposed framework is applicable to distributed sensing systems in which clients correspond to devices, sensing stations, or data-collection sites with heterogeneous acquisition conditions. In such systems, newly deployed devices may provide observations from a shifted domain while previously participating devices continue to supply historically important knowledge. STP-FCL addresses this situation by preserving source-domain prototype semantics on clients and regulating the contribution of domain-shifted updates on the server. The current experiments use visual benchmarks as controlled proxies for distributed sensing scenarios; validation on real sensor streams and heterogeneous sensing hardware remains an important direction for future work.

## 7. Conclusions

In this paper, we studied dynamic-domain spatio-temporal forgetting in federated continual learning, where sequential task learning and dynamically joining new-domain clients jointly degrade source-domain knowledge. We proposed STP-FCL, a coordinated client–server framework that combines prototype-preserved local optimization with domain-projected aggregation to reduce representation drift and update interference. Experiments show that STP-FCL improves source-domain retention and mixed-domain accuracy, with a trade-off in target-domain accuracy. These results support the effectiveness of STP-FCL in preserving aggregate source-domain performance under task evolution and new-domain participation.

## Figures and Tables

**Table 1:** Main results under dynamic-domain federated continual learning. Source AA, Target AA, and Mix AA denote post-joining average accuracy on the source domain, target domain, and mixed observed classes, respectively. Bold and underlined values indicate the best and second-best results in each column, respectively.

Method	MNIST+SVHN			MNIST+SynthDigits		
	Source AA	Target AA	Mix AA	Source AA	Target AA	Mix AA
FedLwF	22.90	21.39	21.64	24.38	17.87	21.20
FedEWC	18.59	21.28	20.53	18.13	21.07	19.57
Re-Fed	17.71	21.52	20.46	19.35	20.60	19.96
FedSpace	50.89	**63.11**	59.72	84.63	30.70	58.28
TagFed	50.76	48.97	49.46	69.49	70.94	70.20
RefFiL	62.95	54.74	57.02	72.81	70.20	71.53
SR-FDIL	59.25	49.74	52.38	70.93	**76.31**	73.56
STP-FCL	**81.36**	57.40	**64.05**	**87.31**	70.21	**78.96**

**Table 2:** Ablation results on the MNIST→SVHN setting over three random seeds (2002, 2003, and 2004). Results are reported as mean ± sample standard deviation (%), with the standard deviation calculated using n−1 in the denominator. The highest mean in each accuracy column is shown in bold.

Case	$L_{\mathrm{proto}}+L_{\mathrm{con}}$	$L_{\mathrm{spa}}$	SAM	DPA	Source AA	Target AA	Mixed AA
1	×	✓	✓	✓	80.33±3.33	57.00±1.63	63.48±1.94
2	✓	×	✓	✓	80.25±1.98	57.06±0.53	63.45±0.89
3	✓	✓	×	✓	81.10±1.09	57.47±1.24	64.03±0.71
4	✓	✓	✓	×	68.69±4.87	61.62±1.67	63.58±0.44
5	✓	✓	✓	✓	82.83±1.29	57.98±1.10	64.87±0.96

**Table 3:** Parameter sensitivity analysis on MNIST+SVHN. Bold values indicate the best results in each column.

Setting	Parameter	Value	Source AA	Mix AA
SPA	$\lambda_{\mathrm{src}}$	1.0	78.32	62.18
	$\lambda_{\mathrm{src}}$	3.0	**81.36**	**64.05**
	$\lambda_{\mathrm{src}}$	5.0	78.78	62.63
DPA	Fusion scale	0.5×	**85.62**	48.04
	Fusion scale	1.0×	81.36	**64.05**
	Fusion scale	1.5×	64.13	63.43

**Table 4:** Communication and computational overhead of all compared methods on the MNIST→SVHN setting. The standard model exchange is shared by all methods and costs approximately 875.20 MiB per communication round with 10 selected clients. “Extra payload” denotes method-specific communication beyond the standard model upload/download.

Method	Extra Payload/Round (MiB)	Main Additional Computation
FedLwF	0	Local distillation with a teacher model
FedEWC	0	Local Fisher estimation and EWC regularization
Re-Fed	0	Local personal-model training for sample selection
FedSpace	0.78	Prototype extraction and temporal model averaging
TagFed	0	Server-side task-aware classifier aggregation
RefFiL	1.76	Prompt generation and server-side prompt clustering
SR-FDIL	2.96	Generator/discriminator-based replay and discriminator aggregation
STP-FCL	1.02	SAM-based local optimization, SPA loss, and server-side DPA

## Data Availability

The datasets analyzed in this study are publicly available. The code will be made available upon reasonable request.

## Appendix A. Asynchronous Class-Incremental Task Streams

For the digit-based experiments, the ten classes are divided into five class-incremental tasks, with two classes assigned to each task. To model task-level heterogeneity across clients, each client *m* is assigned a client-specific task sequence

(A1) $$T_{m}=\left\{T_{m}^{1},T_{m}^{2},\ldots ,T_{m}^{5}\right\}.$$

The class order in $T_{m}$ may differ across clients, while all clients share the same global label universe.

Each client also has client-specific task-switching boundaries ${\left\{b_{m}^{k}\right\}}_{k=1}^{6}$. Client *m* observes task $T_{m}^{k}$ during the communication rounds satisfying

(A2) $$b_{m}^{k}\leq t<b_{m}^{k+1}.$$

For each client, candidate durations for the first four tasks are sampled uniformly from the integers between $\left\lceil 0.7\bar{L}\right\rceil$ and $\left\lfloor 1.3\bar{L}\right\rfloor$, inclusive, where $\bar{L}$ is one fifth of the client’s available training horizon. The fifth duration is the remainder. If this remainder is non-positive, all four candidate durations are resampled until the fifth duration is strictly positive. Consequently, different clients may be learning different tasks during the same communication round.

Source-domain task streams start at the beginning of federated training. For a target-domain client, the first task becomes active only after the client joins the federation. In the primary setting, target-domain clients join at $t_{0}=150$, so their task streams start from that round. This procedure creates task-level heterogeneity independently of the domain shift and is applied consistently to all compared methods.

## Appendix B. Additional Robustness and Resource Analyses

*Cross-Domain Evaluation on PACS*

To evaluate the method under a more challenging visual domain shift, we conduct an additional experiment on the PACS Photo–Sketch setting. The federation contains 20 Photo source-domain clients and 10 Sketch target-domain clients. The two domains share a seven-class label space and follow a class-incremental task sequence of 2+1+1+1+2. Sketch clients become available at round 150, and the client data are partitioned using a power-law Dirichlet distribution with α=4.0. The comparison uses random seed 2002 and includes STP-FCL, FedSpace, and RefFiL.

This single-seed experiment provides preliminary evidence of the source-retention–target-adaptation trade-off on PACS. Compared with RefFiL, STP-FCL achieves higher source-domain accuracy (34.96% versus 17.64%), but lower target-domain accuracy (11.76% versus 18.47%). Its mixed accuracy is only 0.45 percentage points higher (18.68% versus 18.23%). Thus, the observed source-domain advantage is accompanied by reduced target-domain performance.

Because this comparison uses only random seed 2002, it does not establish a statistically reliable improvement in mixed accuracy or robustness across random seeds. We therefore present these results as preliminary, rather than conclusive evidence of effectiveness beyond the digit benchmarks. Multi-seed validation on PACS remains necessary to assess the stability of the observed trade-off.

**Table A1:** Preliminary single-seed results on the PACS Photo–Sketch setting using random seed 2002. All methods follow the same evaluation protocol. Bold values indicate the highest observed accuracy in each column among the compared methods, not statistical significance.

Method	Photo (Source)	Sketch (Target)	Mixed
FedSpace	15.71	12.91	13.75
RefFiL	17.64	**18.47**	18.23
STP-FCL	**34.96**	11.76	**18.68**

### Appendix B.1. Staged Target-Client Participation

We further evaluate robustness when target-domain clients join at different times. In the MNIST→SVHN setting, the ten target-domain clients are divided into three groups with a 4/3/3 split. The three groups join the federation at rounds 100, 150, and 200, respectively. The comparison includes FedSpace, RefFiL, and STP-FCL.

**Table A2:** Results under staged target-client participation on the MNIST→SVHN setting. Bold values indicate the best results in each column.

Method	Source AA	Target AA	Mix AA
FedSpace	52.08	**51.06**	51.34
RefFiL	65.86	50.76	54.95
STP-FCL	**78.79**	50.08	**58.05**

Under staged participation, STP-FCL obtains the highest Source AA and MixAA. This result shows that the proposed framework remains effective when target-domain clients arrive at different stages of training.

### Appendix B.2. Focused Multi-Seed Evaluation

To examine the stability of the main performance trend, we repeated the experiments for STP-FCL and two competitive baselines, FedSpace and RefFiL, using three random seeds: 2002, 2003, and 2004. The values below are reported as mean ± sample standard deviation.

**Table A3:** Focused three-seed evaluation on MNIST+SVHN.

Method	Source AA	Target AA	Mix AA
FedSpace	56.78±5.18	64.59±2.50	62.43±2.68
RefFiL	62.34±4.43	61.24±6.11	61.54±4.89
STP-FCL	82.83±1.29	57.98±1.10	64.87±0.96

**Table A4:** Focused three-seed evaluation on MNIST+SynthDigits.

Method	Source AA	Target AA	Mix AA
FedSpace	84.12±0.80	31.17±0.52	58.14±0.36
RefFiL	76.20±5.60	77.70±7.10	77.60±6.31
STP-FCL	88.60±1.16	72.58±2.05	79.16±1.17

### Appendix B.3. Computational Cost

We additionally measured the local training time and server-side aggregation time under controlled workloads. Local measurements use an NVIDIA RTX 4090, 256 preprocessed SVHN images, a batch size of 64, and 10 local epochs. Each value is the mean ± sample standard deviation over five timing repetitions after warm-up. Method-specific local operations are retained.

**Table A5:** Controlled client-side computation measurements. Prediction-model parameters are reported in millions.

Method	Prediction-Model Parameters (M)	Local Update Time (s)
FedLwF	11.277	0.69±0.02
FedEWC	11.277	1.56±0.04
Re-Fed	11.277	1.04±0.12
FedSpace	11.277	6.74±0.12
TagFed	11.277	7.57±0.35
RefFiL	15.485	7.47±0.45
SR-FDIL	11.277	2.56±0.02
STP-FCL	11.277	16.42±1.36

The additional local computation of STP-FCL is mainly introduced by SAM-based optimization. In the three-seed MNIST+SVHN evaluation, STP-FCL achieves mean Source AA of 82.83% and mean MixAA of 64.87%, compared with 56.78% and 62.43% for FedSpace. These correspond to improvements of 26.05 and 2.44 percentage points, respectively. Thus, under the evaluated protocol, the additional training computation is accompanied by higher mean source-domain and mixed-domain accuracies.

**Table A6:** Server-side aggregation time on an Intel Xeon Platinum 8470Q CPU using four threads. Each call aggregates ten model updates. Values are reported as mean ± sample standard deviation over 20 timing repetitions after three warm-up calls.

Aggregation	Time per Call (ms)
FedAvg	35.69±2.42
FedSpace	53.25±4.50
DPA (aligned updates)	142.41±27.19
DPA (conflicting updates)	162.52±20.68

For conflicting updates, DPA adds approximately 0.109 s per aggregation call relative to FedSpace. SAM operates only during local training and DPA only during server-side aggregation; neither introduces additional inference-stage computation. STP-FCL therefore trades additional continual training computation for stronger source-domain retention and mixed-domain performance while retaining a compact prediction model.

## Appendix C. Additional Analysis of Spatial Forgetting

To directly quantify the immediate effect of target-domain client participation on previously learned source-domain knowledge, we report the instantaneous source-domain accuracy drop. Let $A_{\mathrm{src}}\left(t_{0}^{-}\right)$ denote the source-domain accuracy at the last evaluation point before target-domain clients join, and let $A_{\mathrm{src}}\left(t_{0}^{+}\right)$ denote the accuracy at the first evaluation point after their participation begins. We define the instantaneous spatial forgetting as

(A3) $$F_{\mathrm{spa}}={\left[A_{\mathrm{src}}\left(t_{0}^{-}\right)-A_{\mathrm{src}}\left(t_{0}^{+}\right)\right]}_{+},$$

where the results are reported in percentage points. A larger value indicates a larger immediate degradation of source-domain performance caused by target-domain participation.

Table A7 provides a direct before-and-after measure of the source-domain degradation caused by target-domain participation. On MNIST→SVHN, STP-FCL limits the instantaneous source-domain accuracy drop to 37.33 percentage points, substantially lower than the drops of FedSpace (54.03) and RefFiL (77.73). This result is consistent with the role of source-preserved prototype alignment and domain-projected aggregation in reducing the impact of cross-domain update interference.

**Table A7:** Instantaneous source-domain accuracy drop after target-domain clients join. The drop is measured between the last evaluation point before target-domain participation and the first evaluation point afterward. Lower values indicate less immediate spatial forgetting seed is 2002.

Method	MNIST→SVHN $F_{\mathrm{spa}}$ (Percentage Points)	MNIST→SynthDigits $F_{\mathrm{spa}}$ (Percentage Points)
FedLwF	1.03	1.30
FedEWC	12.00	11.67
Re-Fed	4.50	9.37
FedSpace	54.03	**3.01**
TagFed	47.81	14.02
RefFiL	77.73	21.84
SR-FDIL	33.64	26.01
STP-FCL	**37.33**	14.71

On MNIST→SynthDigits, FedSpace exhibits the smallest instantaneous drop (3.01 percentage points). Nevertheless, STP-FCL limits the drop to 14.71 percentage points, which is substantially lower than those of the domain-incremental baselines RefFiL (21.84) and SR-FDIL (26.01). Moreover, STP-FCL achieves the highest post-joining Source AA and Mixed AA on this setting, as reported in the main results. These results suggest that STP-FCL provides a favorable balance between immediate source-domain retention and post-joining overall performance across the evaluated domain shifts, although the magnitude of instantaneous forgetting varies with the dataset and the compared method.

## Appendix D. Source-Domain Retention at Later Training Rounds

To complement the immediate joining-point accuracy drop, we examine source-domain retention at later training rounds on the MNIST→SVHN setting. The comparison includes FedSpace, RefFiL, and STP-FCL over three random seeds (2002, 2003, and 2004). We evaluate the global model on the same fixed source-domain test set at rounds 149, 150, 250, and 399, keeping the evaluation samples, class set, and prediction protocol unchanged. Round 149 precedes target-domain participation, round 150 corresponds to the first global model incorporating target-domain updates, and round 399 is the final training round.

Let $A_{\mathrm{src},s}^{\mathrm{fixed}}\left(t\right)$ denote the source-domain accuracy, expressed as a percentage, at round *t* for seed *s*. We define the source-domain degradation relative to the pre-joining model as

(A4) $$D_{\mathrm{src},s}\left(t\right)=A_{\mathrm{src},s}^{\mathrm{fixed}}\left(149\right)-A_{\mathrm{src},s}^{\mathrm{fixed}}\left(t\right),\;t\in \left\{150,250,399\right\}.$$

Positive values indicate degradation, whereas negative values indicate improvement over the pre-joining model. Unlike the non-negative immediate-drop metric $F_{\mathrm{spa}}^{\mathrm{imm}}={\left[D_{\mathrm{src}}\left(150\right)\right]}_{+}$, this longitudinal measure retains the sign of the accuracy difference. Degradation is calculated separately for each seed before computing the mean and sample standard deviation across the three runs.

Table A8 reports both the pre-joining accuracy and the subsequent degradation, allowing retention to be assessed relative to each method’s own starting performance. STP-FCL exhibits the smallest mean immediate degradation among the three methods. At round 250, its mean degradation is 3.17 percentage points, compared with 28.12 for FedSpace and 13.87 for RefFiL. At the final round, its mean degradation is 5.48 percentage points, compared with 14.69 and 11.85, respectively. STP-FCL also achieves the highest mean final source-domain accuracy of 86.16%. These results support stronger source-domain retention at the evaluated later rounds, extending the comparison beyond the immediate joining-point drop.

**Table A8:** Source-domain retention on MNIST→SVHN over three random seeds. All evaluations use a fixed source-domain test set. Values are reported as mean ± sample standard deviation. Accuracy is expressed in percent, and degradation relative to round 149 is expressed in percentage points. Bold values indicate the lowest mean degradation or highest mean final accuracy among the compared methods.

Method	Pre-Joining Accuracy	Immediate Degradation $D_{\mathrm{src}}\left(150\right)$	Round-250 Degradation $D_{\mathrm{src}}\left(250\right)$	Final Degradation $D_{\mathrm{src}}\left(399\right)$	Final Accuracy
FedSpace	84.87±6.68	49.45±9.21	28.12±11.00	14.69±5.44	70.17±6.22
RefFiL	85.47±6.17	69.87±8.17	13.87±11.04	11.85±2.91	73.62±3.71
STP-FCL	91.64±0.01	30.54±6.10	3.17±0.97	5.48±1.69	86.16±1.68

This analysis follows a round-based global evaluation perspective used in asynchronous federated continual learning [22]. With client-specific task orders and switching times, there is no shared global task-completion boundary. The reported metric therefore characterizes aggregate source-domain retention under concurrent task evolution and target-domain participation, rather than task-specific temporal forgetting.

## Appendix E. Method-Specific Hyperparameters

To facilitate reproduction, Table A9 summarizes the method-specific hyperparameters used for the compared baselines.

**Table A9:** Method-specific hyperparameters.

Method	Method-Specific Settings
FedLwF	Distillation weight $\lambda_{\mathrm{LwF}}=3.0$; distillation temperature $T_{\mathrm{KD}}=2.0$; FedAvg aggregation.
FedEWC	Regularization weight $\lambda_{\mathrm{EWC}}=1.0$; Fisher-estimation learning rate $10^{-3}$; FedAvg aggregation.
Re-Fed	Replay memory size 1000; $\lambda_{p}=0.1$; personal-model learning rate $10^{-3}$; 5 personal-model training epochs.
FedSpace	Prototype replay weight $10^{-2}$; representation regularization weight $10^{-2}$; prototype exponential-moving-average coefficient 0.1; temporal fusion weight 0.5.
TagFed (adapted)	weight decay $2\times 10^{-4}$; logit temperature τ=0.1; previous-global-model fusion weight 0.5.
RefFiL	initial contrastive temperature τ=0.9; minimum temperature $\tau_{\min}=0.3$; temperature-decay parameters γ=0.1 and β=0.05; CE/GPL/DPCL loss weights 1/1/1.
SR-FDIL	Exemplar memory size M=500; $\lambda_{\mathrm{SR}}=0.5$; GAN learning rate $10^{-3}$;
